# Toward a Detailed Evaluation of Wireless Industrial Data Distribution Approaches †

**DOI:** 10.3390/s22072533

**Published:** 2022-03-25

**Authors:** Theofanis P. Raptis, Andrea Formica, Elena Pagani, Andrea Passarella, Marco Conti

**Affiliations:** 1 Institute of Informatics and Telematics, National Research Council, 56124 Pisa, Italy; andrea.passarella@iit.cnr.it (A.P.); marco.conti@iit.cnr.it (M.C.); 2 Department of Computer Science, University of Milan, 20133 Milan, Italy; andrea.formica@studenti.unimi.it (A.F.); elena.pagani@unimi.it (E.P.)

**Keywords:** data distribution, OMNeT++, Industry 4.0, WirelessHART, RPL

## Abstract

Data distribution is a cornerstone of efficient automation for intelligent machines in Industry 4.0. Although in the recent literature there have been several comparisons of relevant methods, we identify that most of those comparisons are either theoretical or based on abstract simulation tools, unable to uncover the specific, detailed impacts of the methods to the underlying networking infrastructure. In this respect, as a first contribution of this paper, we develop more detailed and fine-tuned solutions for robust data distribution in smart factories on stationary and mobile scenarios of wireless industrial networking. Using the technological enablers of WirelessHART, RPL and the methodological enabler of proxy selection as building blocks, we compose the protocol stacks of four different methods (both centralized and decentralized) for data distribution in wireless industrial networks over the IEEE 802.15.4 physical layer. We implement the presented methods in the highly detailed OMNeT++ simulation environment and we evaluate their performance via an extensive simulation analysis. Interestingly enough, we demonstrate that the careful selection of a limited set of proxies for data caching in the network can lead to an increased data delivery success rate and low data access latency. Next, we describe two test cases demonstrated in an industrial smart factory environment. First, we show the collaboration between robotic elements and wireless data services. Second, we show the integration with an industrial fog node which controls the shop-floor devices. We report selected results in much larger scales, obtained via simulations.

## 1. Introduction

Industry 4.0 is becoming characterized, among others, by increasing levels of automation achieved by the collaboration between robots and more in general intelligent machines. Each physical machine is likely to embed a higher and higher number of devices (e.g., industrial IoT devices) constantly generating data, to be exchanged and exploited for controlling the collective behavior of the smart factory. In this regard, optimization of the data distribution process is a major objective for industry [1]. Intelligent industrial environments operate using advanced sensors and communication technologies; therefore, large amounts of data are generated and collected in shop-floors, industrial fields and enterprise facilities, necessitating data handling technologies to provide integrated environments where the production services can run efficiently and be administered effectively [2].

The importance of data in Industry 4.0 deployments is becoming increasingly relevant. Industrial use cases produce greater amounts of data, both at the node level (for example, AV IoT devices) and at the system level, due to the augmenting numbers of devices collaborating with industrial and robotic modules. Furthermore, many key Industry 4.0 applications are fused via effective data administration across networked elements. Consequently, a recently adopted approach is to view industrial networks as data-centric and to include efficient data administration as a top priority in such networking settings [3]. In the recent past, pure cloud computing was providing us with an opportunity to address emerging data-centric problems seamlessly at a central point of control [4]. In many cases, hierarchy clouds or fog-oriented approaches have also been used for providing additional usability [5]. Although there have been approaches in the literature about storing and accessing industrially relevant data on the cloud, the additional cost of moving the data among local and distant locations has already been shown as high for some application scenarios and prohibiting for others [6].

When the Industry 4.0 paradigm was born, there were no standards capable of meeting all fundamental requirements. Since the third industrial revolution, in the industries there were connected machines, but they only worked with Ethernet, which cannot guarantee the respect of all the constraints, in particular having a short and deterministic latency for a multitude of devices. The first attempt that was made to overcome the problem was the introduction of the Real Time Ethernet protocol [7] which works with common network devices (switches, bridges and routers) and guarantees deterministic latency, but there have been developed numerous incompatible implementations. Determinism cannot be guaranteed in networks that use Transmission Control Protocol (TCP) as the transport layer protocol in the TCP/IP stack as it involves retransmissions when messages are lost and this takes time. In addition, Real Time Ethernet generates a lot of traffic to send little data as messages must be encapsulated in Ethernet frames that are 64 bytes in size. The second solution proposed was Time-Sensitive Networking (TSN) [8], whose main features are synchronization among nodes, traffic shaping, the existence of shared rules for path selection and resistance to failures. The problem of TSN is that it is not a deterministic protocol, but as mentioned above, this is one of the main requirements and therefore it is not suitable for use in Industry 4.0.

Ethernet is not suitable also because in an industrial environment, there can be hundreds if not thousands of nodes, and if they were all wired, it would create an environment in which it would be difficult to ensure the safety of workers. Furthermore, it is difficult to imagine the use of a mobile system connected to the Internet via a cable since the length of the cable would limit its movement. Finally, wiring the whole network would be an expensive operation and in some cases it could even be difficult to connect the nodes to a cable because they are located in places that are not easily accessible. Given these reasons, it is easy to understand the choice to work with wireless technologies in the industrial field. The main protocols for wireless communication are Near Field Communication (NFC) [9] and Radio Frequency Identification (RFID) [10], but they are not always suitable because they have a very small radio range. Bluetooth has a better radio range (about ten meters) but creates small networks (up to 255 devices connected to the same master, but only 7 can be active and communicate with the master); it supports only the star topology and not uses IP for addressing. WiFi has a greater radio range but it is not economic from the point of view of energy consumption. IEEE has developed, however, IEEE 802.15.4, a protocol for Low-Power Wireless Personal Area Networks. This protocol has driven the design of many related wireless standards, tailored to industrial communication requirements, such as ZigBee [11], WIA-PA [12], ISA100 Wireless [13] and WirelessHART [14]. The last one is of interest to our current study.

The number of technologies to be integrated in order to implement a smart data distribution system is large. Current data management solutions primarily consider centralized data management approaches, where all data are collected at a central location (usually in some cloud platform) and then distributed across nodes when needed. However, due to the expected increase in the data generation rate by devices, coupled with the stringent requirements on data delivery for control processes, more distributed solutions are going to be needed, where data are stored on devices closer to where they are needed and delivered directly from there. In addition, robust solutions are also needed, exploiting all types of wireless communication technologies available, in order to reduce the impact of interference, malfunctioning or unavailability of each specific technology.

### 1.1. Our Contribution

We propose decentralized data distribution approaches supported by a hierarchical and multi-tier network structure [15]. The proposed solution combines local and decentralized management with centralized decisions to efficiently use the available network resources and meet the requirements of Industry 4.0 applications. Contrary to the recent theoretical and abstract simulative comparisons, we

1. Analyze the characteristics of four protocol stacks for data distribution in wireless industrial networks, built mainly from proposed standards such as IEEE 802.15.4 [16], IETF RPL [17] and IEC WirelessHART [18], and adopt either a centralized (data accessed only at the central network controller) or distributed (data replicated in *proxies*) approach. We implement the methods in OMNeT++ Network Simulator by extending and customizing the simulator according to the needs of our study. We evaluate the performance of the four methods via a detailed simulation analysis. The use of OMNeT++ reveals detailed performance insights about industrial data distribution. We show that a limited number of data *proxies*—carefully placed in order to limit delay in data retrieval—effectively helps in fulfilling latency constraints.
2. We implement two test cases so as to highlight the applicability of (mobile and stationary) distributed lightweight data management within an actual industrial environment. In order to demonstrate how we can apply the data management concepts and showcase their feasibility, we designed and implemented a small-scale demonstration at the premises of a smart manufacturing experimentation facility (IK4-Tekniker), requiring robust data exchange between a heterogeneous set of autonomous factory machines. The core of this concept is to demonstrate how we can use smart data distribution at the manufacturing experimentation facility so as to achieve cost-effective operations, fault tolerance and dynamic, plug-and-play smart data distribution solutions.

### 1.2. Roadmap of the Paper

The rest of the paper is organized as follows. In Section 2, we present the most related recent works in the literature and highlight our contribution beyond the state of the art. In Section 3, we present the methods for industrial data distribution that we consider in this paper and provide a description of their building blocks. In Section 4, we present the developed simulation model and the related route computation and channel scheduling processes. In Section 5, we present the performance results of the presented methods in two different scenarios: one with a variable number of nodes and fixed percentage of producers and consumers and one with a fixed number of nodes and variable number of consumers. In Section 6, we describe the two test cases demonstrated in an industrial smart factory environment, and we show the integration with an industrial fog node which controls the shop-floor devices. We report selected results in much larger scales, obtained via simulations. Finally, in Section 7, Section 8 and Section 9, we present some open problems, future perspectives and conclusions, respectively.

## 2. Related Works

Regarding our first contribution, in the recent literature, there have been several comparisons (both theoretical and simulative) among enablers that we consider.

### 2.1. Theoretical and Methodological Contributions

- A comparison of WirelessHART and ZigBee is provided in [19]. The authors explain why ZigBee is not the most appropriate protocol for use in many industrial use cases. They claim that the protocol was missing some characteristic features, which gave ground to the development of other protocols and standards such as WirelessHART. The authors also demonstrate the design directives that render WirelessHART more appropriate for industrial use cases, constraints and requirements.
- A comparison of WirelessHART and ISA100 Wireless, both from a technical and a systematical point of view, is provided in [20]. The authors presnet the inherent strengths and weaknesses of the two protocols and how these influence their suitability for different industrial use cases. The authors conclude that both protocols are capable of robust and reliable communication in harsh industrial environments. As a result, predicting which protocol will emerge as the de facto standard for industrial use cases in the shop-floor is very difficult.
- A theoretical study on the relationship between WirelessHART and IEEE 802.15.4e, as well as on what impact the latest IEEE 802.15.4e has on WirelessHART and how industrial operators could benefit from both standards, is provided in [21].

To the best of our knowledge, there is no direct performance comparison of methods incorporating all three aforementioned elements (WirelessHART, RPL, proxies), and in most cases—the opposite of our approach—the comparisons are purely theoretical ([19,20,21]). Those observations lead us to the conclusion that there is a research gap in the litarature which refers to practical comparisons of WirelessHART, RPL and proxy-employing methods.

### 2.2. Simulation Modeling Contributions

- An RPL comparison to another routing protocol called LRP is provided in [22]. The authors compare RPL and LRP in small-scale deployments. They measure the impact of assymetric links on the chosen protocols and demonstrate the features of LRP to deal with such settings. They also demonstrate the behavior of both protocols when the active links vary over time.
- An RPL comparison to another routing protocol called LOADng is provided in [23]. The authors compare the performance of the two protocols under heterogenous traffic patterns in realistic non-sparse non-uniform network deployments. They use the Cooja simulator and the the ContikiRPL implementation and an implementation of their LOADng.
- A latency-aware proxy selection method comparison to RPL is provided in our past paper [24]. In this paper, we exploit distributed data management to overcome the following issue: Given a set of data, the set of consumer nodes and the maximum access latency that consumers can tolerate, we consider a method for identifying and selecting a limited set of proxies in the network where data needed by the consumer nodes can be cached. We evaluate its performance using in large-scale deployments.

To the best of our knowledge, the majority of such simulation modeling contributions employ high-level simulation tools (such as Matlab) and are therefore not able to uncover in-depth the impact that each method has on the underlying networking settings. This observation leads us to the conclusion that there is a research gap in the literature which refers to accurate, fine-grained simulation modeling of WirelessHART and proxy employing methods.

### 2.3. On-Site Deployment Evaluations

The on-site deployments within factories are not uncommon. The extent of the deployments, as well as the selected technologies, greatly vary. For example:

- In [25], the authors present a network setting which is built in a collaborative robotics experimental facility. The system wirelessly connects a dual-arm robot and a mobile robot that collects and supplies components to the dual-arm robot.
- In [26], the authors explore the communication quality achieved by TSCH in a setup that includes real motes exposed to a realistic interfering industrial traffic.
- In [27], the authors introduce the design of a general yet accurate and reproducible measurement setup that will be exploited to assess the performance of the main open-source industrial implementations.
- Last but not least, in [28], the authors explore the impact of rate adaptation algorithms on Wi-Fi-based factory automation systems.

Although the above works are valuable in their own implementation limits, they select isolated industrial application concepts. This observation leads us to the conclusion that there is a research gap in the literature which refers to the focus on deployments (of both stationary and mobile scenarios) with IEEE 802.15.4 combined with fog nodes and robotic elements.

Table 1 is displaying a comparison among the reported works and highlighting the main contributions of this paper.

## 3. Methods for Industrial Data Distribution

In this section, we design four methods targeting data distribution for wireless industrial networks. We first present the network model we consider, then the technological and methodological building blocks that we use and finally the centralized and decentralized methods that we design.

We consider networks of industrial devices which consist of sensor motes and actuators and are operating at the physical layer in the 2.4 GHz ISM band using IEEE 802.15.4 radios; this choice is in accordance with the operating conditions of WirelessHART, thus guaranteeing fair comparison among the analyzed methods. In network configurations of such type, a central network controller (CNC) maintains centralized network knowledge. This is usual in industrial applications, in which the locations of the nodes are known, traffic flows are deterministic and communication patterns are established a priori. We assume that the CNC knows all the shortest paths in the network. This is in accordance with the functionalities in charge to the 6LBRs (6LoWPAN area border routers) in the 6TiSCH architecture for industrial networks.

Sensor nodes perform monitoring tasks (producers), and in some cases, their sensor data are needed either by other sensor nodes, which could need additional data to complement their own local measurement, or by actuator nodes, which use the sensor data so as to perform an actuation (consumers). When needed, a consumer can ask for data of interest from a sensor node using the primitives defined by the specific industrial application; application messages are forwarded to their destinations by leveraging the service provided by the underlying routing protocol. As said, the CNC is in charge of the control plan managing the data distribution process. However, the actual data distribution may be decentralized and cooperative by delegating to identified nodes in the network (*proxies*) the data caching. Proxy nodes are able to store data that can be accessed in a timely manner from the consumer nodes of the network.

We assume that the data generation and access processes are not synchronized. Specifically, we assume that data consumers request data at an unspecified point in time after data have been generated by data producers. This demarcated model of data exchanges can be formulated as a publish–subscribe (pub/sub) model [29]. In a pub/sub model, a consumer subscribes to data, i.e., denotes interest for it to the corresponding proxy, and the relevant producer publishes advertisements to this proxy. The pub/sub process is regulated at the CNC, which maintains knowledge on the sets of producers, consumers and requests. Based on this, the CNC can find an appropriate set of proxies based on the algorithm we present next. Inside the network, the proxies are responsible for matching subscriptions with publications, i.e., they provide a rendezvous function for storing the available data according to the corresponding subscriptions. The producers do not hold references to the consumers, neither do they know how many consumers are receiving their generated data.

### 3.1. Building Blocks

We now present the main technological and methodological enablers which we use as building blocks for our methods. The layout of the methods is presented in Table 2.

#### 3.1.1. WirelessHART (IEC 62591)

WirelessHART is a wireless sensor networking technology based on the Highway Addressable Remote Transducer Protocol (HART). Developed as a multi-vendor, interoperable wireless standard, WirelessHART was defined for the requirements of industrial field device networks. The protocol utilizes a time-synchronized, self-organizing and self-healing mesh architecture. An important characteristic of the WirelessHART MAC layer which differentiates it from the typical 802.15.4 CSMA/CA MAC layer is that it combines slow frequency-hopping and a TDMA scheme that utilizes a centralized a priori slot allocation mechanism [30]. Two different mechanisms are provided for message routing: graph routing and source routing. In this work, we use the source routing option, which uses ad hoc created routes for the messages (instead of the pre-determined, redundant routes of graph routing) without providing any path diversity.

#### 3.1.2. RPL (IETF RFC 6550)

The RPL routing framework is suitable for networks composed of many embedded devices with limited power, memory and processing resources and is able to meet the requirements of a wide range of monitoring and industrial applications. It is widely considered an important networking solution for various industrial sectors. There are two RPL modes: one is called non-storing mode and the other is called storing mode [31]. In non-storing mode, only the routing tree root (in our case the CNC) collects and maintains topology information of the whole network. In storing mode, every node keeps a complete list of routing entries for nodes in its sub-routing trees. Consequently, there is a trade-off between storing and non-storing mode of operation in terms of computing and communication resources. For example, storing mode requires more memory in intermediate nodes while non-storing mode causes data packets to increase in size, thus consuming more transmission power and bandwidth.

#### 3.1.3. Latency-Aware Proxy Selection

In the context of wireless industrial networks, one could leverage the set of nodes present at the edge of the network to distribute functions that are currently being implemented by the CNC. Many flavors of distributed data management exist in the networking literature, depending on which edge devices are used. In [24], the authors consider a distributed approach and use the multitude of sensor nodes present in an industrial physical environment (e.g., a specific factory) to implement a decentralized data distribution, whereby sensor nodes cache data they produce and provide these data to each other upon request. In this case, the choice of the sensor nodes where data are cached must be made to guarantee a maximum delivery latency to nodes requesting those data. The objective function of the proxy selection minimizes the number of proxies. The main constraint is put on the guarantee that the value of the average end-to-end data access latency by the consumers in the network has to remain below a given time threshold imposed by the industrial operator. Each node has to be assigned to one and only one proxy, and all nodes are considered for potentially being selected as proxies.

### 3.2. Centralized Methods for Industrial Data Distribution

The composition of the two centralized methods is shown in Table 2 labeled as C1 and C2. Method C1 is operating as follows: publish–subscribe on the application level, RPL in non storing mode on the network level, CSMA/CA on the MAC level, and IEEE 802.15.4 on the physical level. Method C2 on the other hand is operating as follows: publish–subscribe on the application level, source routing on the network level, WirelessHART on the MAC level and IEEE 802.15.4 on the physical level.

The common aspect of the centralized methods is that they cache the generated data at the CNC. Consequently, when a consumer node requires data access, the data request has to be sent to the CNC and the CNC has to make the data available to the consumer. The main differences between the two centralized methods are in the MAC and NET layers. Method C1 uses RPL in non-storing mode in order to route the relevant data over the typical CSMA/CA protocol of 802.15.4. That means that the slots in each frame are generally contention-based. By contrast, method C2 uses source routing over the WirelessHART MAC protocol. In this case, the use of TDMA and pre-scheduled timeslots prevents message collisions, and frequency hopping and retransmissions limit the effects of temporal and frequency interference (a retransmission occurs on a different frequency).

### 3.3. Decentralized Methods for Industrial Data Distribution

The composition of the two decentralized methods is shown in Table 2, labeled as D1 and D2. Method D1 is operating as follows: publish–subscribe on the application level, RPL in storing mode on the network level, CSMA/CA on the MAC level and IEEE 802.15.4 on the physical level. Method D2 on the other hand is operating as follows: publish–subscribe on the application level, source routing on the network level, WirelessHART on the MAC level and IEEE 802.15.4 on the physical level.

The common aspect of the decentralized methods is that they cache the generated data not at the CNC, but at selected nodes, distributed in the network. Method D1 caches the data at the locations of the lowest common ancestors (LCAs) for each producer–consumer pair, while method D2 caches the data at the locations of the proxies. The proxies are selected before the industrial data distribution process begins, in the following manner: the CNC is initially set as the first proxy of the network. Then, the number of proxies is gradually increased, until it reaches a number such that the average access latency does not violate the maximum data access latency threshold. In every iteration, the exact selection of the next proxy in the network is performed using a myopic greedy addition. Each candidate node is examined, and the one whose addition to the current solution reduces the average access latency the most is added to the incumbent solution. To this end, the latency between a candidate proxy and a consumer is estimated as the length of the shortest path that is connecting them multiplied by the expected latency on each hop. The NET layers of the two methods are different. Method D1 uses RPL in storing mode in order to route the relevant data to the LCAs, while method D2 uses source routing to the proxies.

## 4. Simulation Model

**Figure 1:**
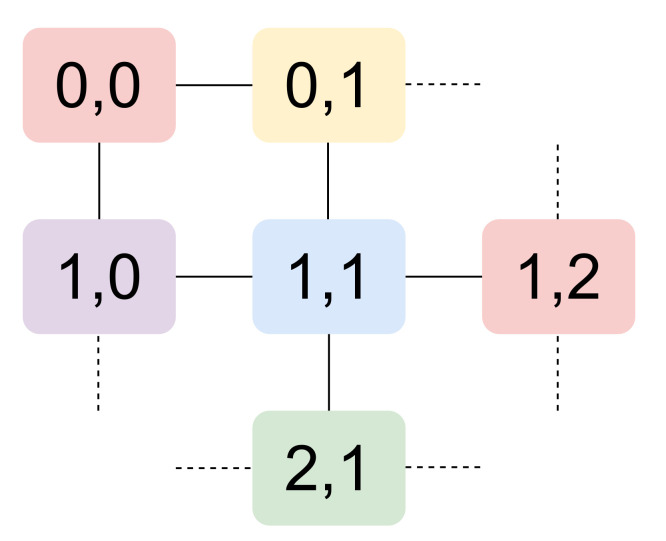
Square grid topology scheme.

We conducted a performance comparison among the methods described in Section 3 through simulations. We used OMNET++ Network Simulator version 4.4.1. https://omnetpp.org/ (accessed on 21 January 2022). As far as the modeling of the WirelessHART protocol stack is concerned, we started with NIST modules [32], https://github.com/usnistgov/tesim_omnetpp (accessed on 21 February 2022) which we customized according to our needs. In this section, we describe the main peculiarities of the models we developed for the presented methods. As a preliminary consideration, we point out that all our simulations consider networks with a square grid topology (Figure 1), where the CNC is always the node with coordinates (0,0) in the top left vertex. We did not reproduce node failures nor battery discharge; hence, all nodes correctly work for the whole simulation duration.

### 4.1. Route Computation

**Figure 2:**
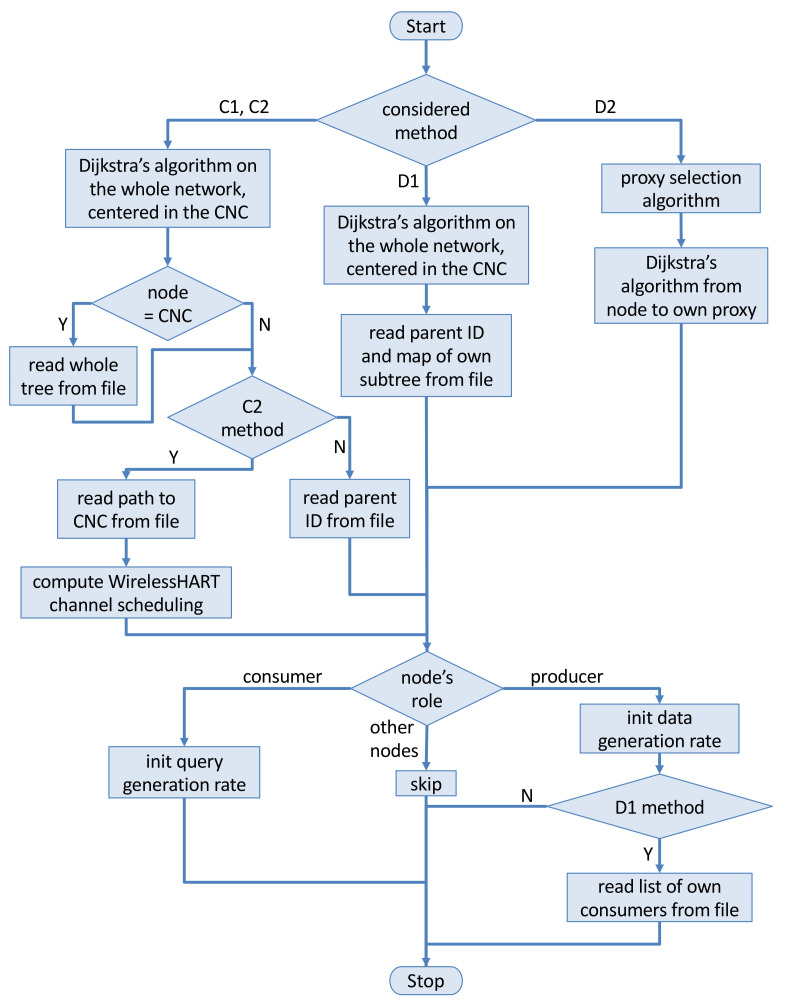
Flowchart representing the adopted methodology.

Since in our simulations nodes never crash and the topology does not change, routes in the networks may be computed offline and then uploaded in the models in order to speed up simulation execution. We consider full-duplex links. As the routing algorithm, we used Dijkstra’s algorithm [33]. With methods C1, C2 and D1, we compute the shortest-path tree rooted in the CNC and connecting all nodes. The computed tree is used to obtain the needed routes in the various methods (see Figure 2).

In RPL non-storing (C1), the routing tables of all nodes but the CNC contain just the address of the parent node in Dijkstra’s tree, while the CNC has routes to every other node and uses them to perform source routing.

In WirelessHART (C2), the CNC node holds the unique data cache. All nodes know only the upstream route in the Dijkstra’s tree to reach the CNC, to which both data and requests are addressed. By contrast, the CNC knows the routes to every other node, used to forward replies.

For RPL in storing mode (D1), the path between any two nodes *s* and *d* follows the branches of Dijkstra’s tree from *s* up to the LCA of both *s* and *d* and then downward along the appropriate branch toward *d*. Hence, every node has in its routing table the routes to both its parent and all its descendants. The determination of the LCA is performed at run time by nodes: producers are preliminarily fed offline with the list of their consumers (bottom right part of the flowchart). Whenever a data item is generated, its producer *s* indicates in the message the list of consumers it is addressed to and forwards it upstream. Each upstream node checks whether it has one (or more) of the listed consumers among its own descendants; if this is the case, the node is the LCA for *s*, and consumer *d* and takes charge of message forwarding downstream toward *d*. It removes *d* from the message header: if the list becomes empty, then the message is discarded; otherwise, a copy of it is forwarded upstream. If a traversed node is itself a targeted consumer, it passes the message to its Application layer and removes its own ID from the list of destinations. If all consumers are in *s*’ subtree, then *s* performs downward forwarding. Finally, in proxy source routing (D2), the proxy selection algorithm is preliminarily run. Afterward, Dijkstra’s algorithm is used to compute the best route from every node to its own proxy. In all cases, the routing tables are filled as a preliminary operation when the simulation starts.

In order to conceptualize the adopted methodology, Figure 2 depicts the steps followed to implement the four analyzed methods and in particular the preliminary computation of the network settings. The top part of the figure concerns the routes computation depending on the considered method; Section 4.1 discusses in detail the adopted policies. The mechanism used in C2 to decide WirelessHART channel scheduling is discussed in Section 4.2. The bottom part of the flowchart supplies further details about settings for specific node categories.

The flowchart must be taken as reference in the following subsections.

### 4.2. Channel Scheduling for WirelessHART

One main problem we encountered in modeling WirelessHART is that the channel scheduling mechanism has yet to be standardized, and just some requirements are indicated in the standard document [34]. Two problems have been dealt with: how to assign slots to pairs of nodes in order to avoid collisions and how to form the superframe.

As far as the former problem is concerned, we implemented a graph coloring algorithm on the tree topology with two constraints to be fulfilled. Considering the maximum number of neighbors owned by inner nodes, five colors are needed for the solution, and they must be assigned to nodes so that two neighbor nodes have different colors, so as to send their frames in different slots and avoid collisions between their transmissions. Moreover, the neighbors of a certain node *n* must have different colors in order to avoid the hidden terminal problem. The example coloring in Figure 1 satisfies both requirements; e.g., contemporary transmissions from nodes with coordinates (0,0) and (1,1) cannot occur, thus avoiding collisions in nodes with coordinates (0,1) and (1,0). Graph coloring is computed offline as well, and assigned slots are configured in the nodes when the simulation starts.

As far as the latter problem is concerned, we opted for assigning slots in the superframe with the aim of maximizing the sleep time of nodes and minimizing latency. The Trivial solution of building a superframe formed by *N* slots, with *N* the number of nodes in the network, is unsatisfactory. Indeed, it neglects the possibility of nodes that contemporarily use a slot of a given color without provoking collisions. This is for instance the case of nodes with coordinates (0,0) and (1,2) in Figure 1, which are not neighbors, nor do they have any neighbor in common. Moreover, it forces a node with *k* neighbors to use *k* superframes to send one frame to each one of them. As an example, in a network with 180 nodes and with slot duration of 10 ms, a node with four neighbors will spend 180×4=720 ms to send a frame to each one of its neighbors. A node stays awake for up to five slots (one for transmission and four for reception to/from neighbors) in each superframe. The alternative solution of having one slot per color in a superframe is disadvantageous under the point of view of energy consumption. Indeed, a node with four neighbors must stay awake in all five slots of the superframe to both send in its slot and receive from its neighbors.

**Figure 3:**
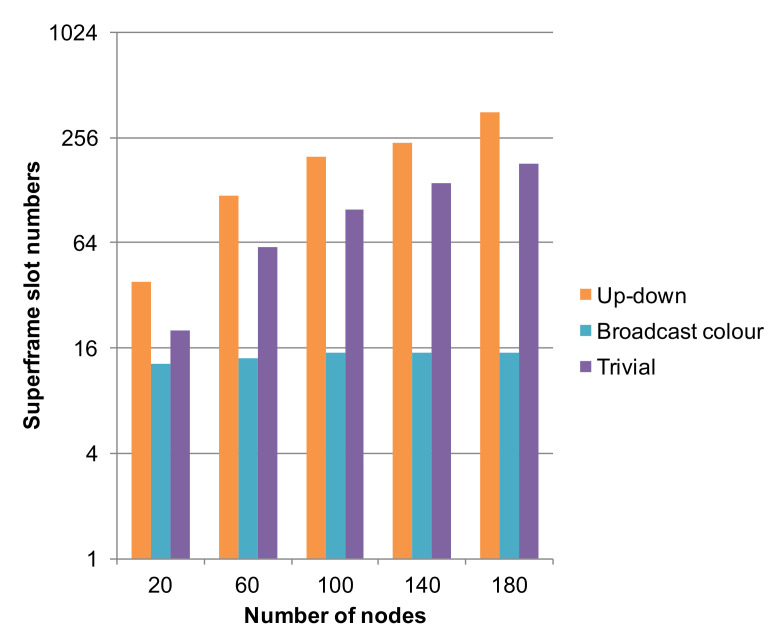
Superframe slot numbers.

In our solution (we indicate it as em Broadcast), by contrast, a superframe is such that, for each node, there are as many slots as the number of its neighbors; e.g., node (1,1) in Figure 1 is entitled to use four blue slots in each superframe. This way, in a certain superframe, a node *n* may send a frame to each one of its neighbors, and parallelism between distant nodes is allowed. With this policy, the upper bound on the superframe size is #colors×node_degree, which in our networks amounts to 5×4=20 slots—corresponding to 200 ms—thus being independent of the network size *N*. The minimum superframe length is achieved when the tree degenerates to a list: in this case, three colors are enough, each node has two neighbors, and 3×2=6 slots per superframe are needed. Hence, latency is limited. Furthermore, this solution allows to save battery energy with respect to the choice of using one slot per color. In fact, a node stays awake for at most eight slots per superframe (four for transmission and four for reception to/from neighbors), if it owns four neighbors. As shown in Figure 3, this is seldom the case in our simulations: with 180 nodes, we obtained a superframe size of 15 slots, and the maximum node degree is 3 in the CNC-centered Dijkstra’s tree. The *Broadcast* solution showed a drawback with the C2 model: it supplies low latencies in uplink paths toward the CNC, while downlink paths show worse delays since up to one superframe is needed to perform one hop. For this reason, we investigated the behavior of alternative coloring policies such as those proposed in [35] trying to build superframes optimized for both path traversal directions. In this case, we obtained far larger superframe sizes (as shown in Figure 2, *Up-down* data series), which might negatively affect latencies. Since we consider the requirements of an industrial environment, we finally resorted to use the *Broadcast* policy in our simulations. We modeled queues in the MAC layer as multiple FIFO queues, one for each destination, so that a node may send a frame to a neighbor as soon as a slot for that neighbor occurs, also if older frames to other neighbors exist (but in the respective, different, queue).

In the next section, the simulation conditions are detailed, including the number of consumers and proxies configured for each simulation.

## 5. Performance Evaluation

We study the performance of the presented methods in two different scenarios, namely, either with variable number of nodes and fixed percentage of both producers and consumers, or with fixed number of nodes and variable number of consumers.

In all simulations, the producers generate new data according to a uniform distribution probability with a period of 3 s. Each consumer generates 400 requests; generation begins with 30 s of delay from the simulation start in order to allow producers to produce some data. Each simulation reproduces 40 min of system operation. Every experiment is run five times, with different random seeds, and results are averaged; in the plots, confidence intervals are also shown. Some preliminary tuning was needed in order to optimize system behavior. We set the queue length at MAC layer to 500, in order to minimize the message loss probability when the network is overloaded. As far as CSMA/CA is concerned, preliminary experiments were allowed to reach 100% message delivery when up to seven retransmissions could be performed in order to overcome collisions. As far as the proxy selection algorithm is concerned, we set the average data access latency threshold to 60 ms.

The main considered performance metrics are: *success rate*, defined as the percentage of requests from consumers that obtained a response; and *latency*, defined as the time elapsed between a request generation by a consumer and the reception of the corresponding response.

**Figure 4:**
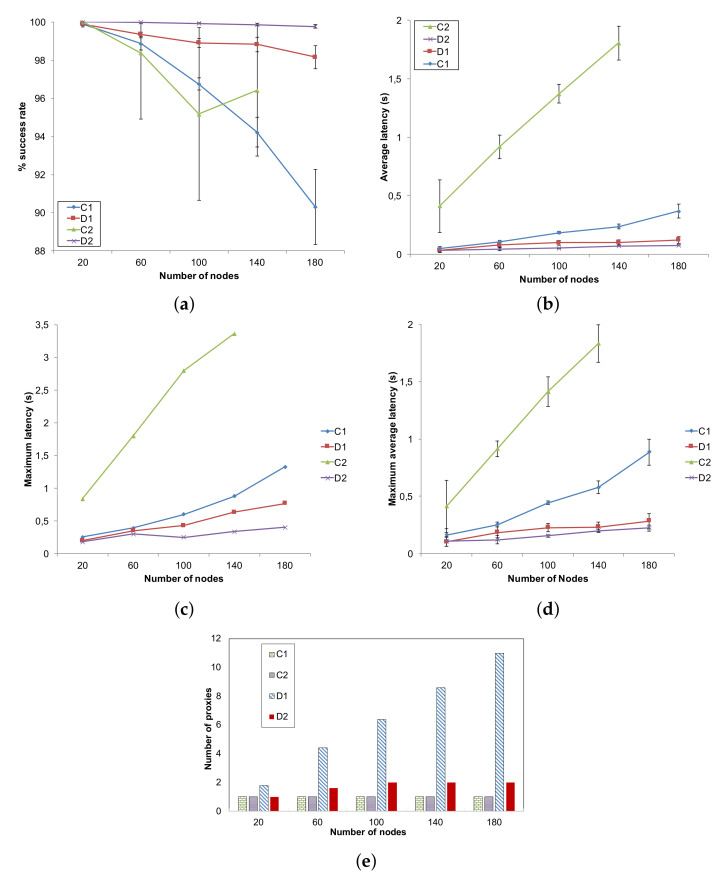
Performance for variable number of nodes. (**a**) Success rate; (**b**) Average latency; (**c**) Maximum latency; (**d**) Maximum average latency; (**e**) Number of proxies.

In the first scenario, the number of nodes varies between 20 and 180. For all cases, both producers and consumers amount to 10% of the total number of nodes. In Figure 4a, the success rate is shown. The use of proxies in method D2 allows to maintain a success rate very close to 100%, thus indicating that the proxy selection algorithm effectively characterizes appropriate number and locations for the nodes selected to cache data items. D1 mimics quite well this behavior thanks to the proxies placed in the LCAs of the producer–consumer pairs; however, it is penalized by traffic congestion at the LCAs and along the tree branches, which causes a higher number of collisions. We recall that the paths used in D1 and D2 are different (see Section 4.1 and Figure 2): while D1 uses a CNC-centered tree, D2 uses optimal paths between producers/consumers and their proxies, thus achieving a better traffic distribution and load balancing in the network, which reduces the probability of collisions. By contrast, centralized solutions are heavily penalized with the growing number of nodes and therefore of both consumers and producers: this leads to a higher number of generated messages that converge toward the CNC. In the case of C1 (RPL in non-storing mode), an increasing number of losses due to collisions is observed as a consequence. In the case of C2 (WirelessHART), which uses time slotting, collisions do not occur. Rather, messages accumulate in the queues and are lost due to buffer overflow, while nodes wait the appropriate slot for transmitting; this effect is more evident as nodes are nearer to the CNC. This also explains the discontinuity for 100 nodes: with increasing number of nodes, the tree produced by the Dijkstra’s algorithm changes, and buffer congestion distributes differently across nodes. Results for C2 with 180 nodes are not available as the model was computationally too heavy for the simulator, which ran out of memory.

The average latency, maximum latency and maximum average latency, which are shown in Figure 4b–d, respectively, confirm that the adoption of a distributed approach allows for reducing latency. The D2 method exceeds D1 in spite of using fewer proxies (see Figure 4e) because in the former, the proxies are chosen on purpose so as to minimize their distance from the consumers, while in the latter they just happen to coincide with the LCAs of the producer/consumer pairs in the Dijkstra’s tree. Indeed, as far as the requirement about latency is concerned, method D1 fulfills it for a 20-nodes network only, while D2 fulfills it quite always adapting to the network: average latency is below the set data access latency threshold of 60 ms for networks of up to 100 nodes. For increasing number of nodes, the constraint is slightly violated (average latency is 77 ms with 180 nodes) and the algorithm tries to deal with this by increasing the number of proxies from 1 to 2, as shown in Figure 4e where the number of LCAs used as proxies by D1 is shown as well (both C1 and C2 have just one proxy that is the CNC).

What was remarked above about WirelessHART leads one to expect that, due to unpredictable queue lengths, it is not true that a scheduled channel access is able to supply a predictable, deterministic latency. Indeed, for C2, the messages waiting in the queues for long times before being transmitted provoke higher delays than the retransmissions performed by CSMA/CA in the case of C1. C1 fulfills the constraint about average latency for a 20-node network, while violates it with higher number of nodes. We also measured the maximum latency observed by consumers: it shows the same trend as the average latency, with a greater separation among curves.

**Figure 5:**
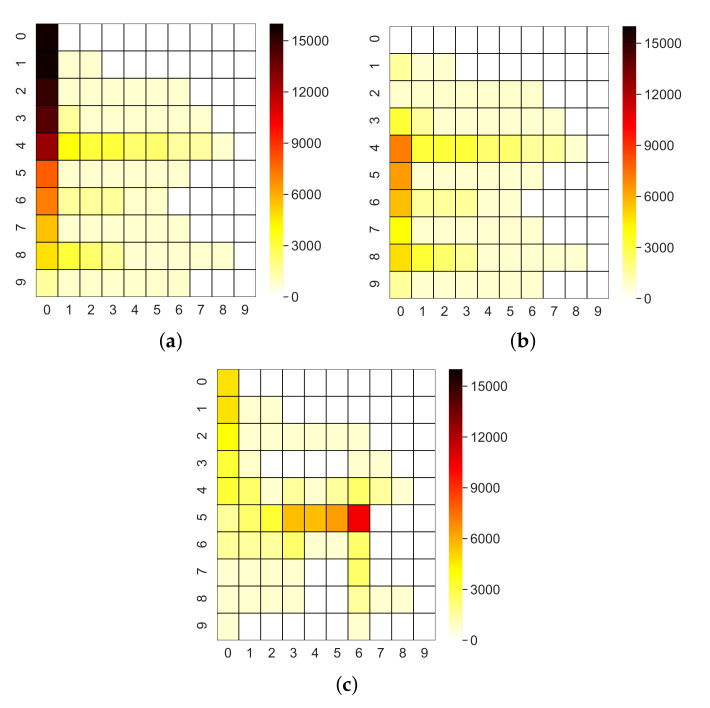
Traffic heatmaps. (**a**) C1 (RPL in non-storing mode); (**b**) D1 (RPL in storing mode); (**c**) D2 (proxy selection algorithm).

In order to study the traffic distribution and also to estimate energy consumption by different nodes, we analyze heatmaps (Figure 5) taken in a network of 100 nodes and based on the number of messages transmitted and received by nodes, which are the most energy-consuming operations.

A centralized solution such as C1 concentrates traffic on the nodes of the tree nearer to the CNC (Figure 5a). As discussed in Section 4 (see also Figure 2), method C2 uses the same Dijkstra’s tree as method C1. Hence, in C2, the traffic distribution—with the same producers and consumers parameters—is equal to that of C1 and the two heatmaps are identical. However, the number of managed messages, compared against the maximum queue length, coupled with the different channel access policy which affects queue lengths, allows to highlight the problem of buffer overflow mentioned above for C2: nodes nearer to the CNC discard almost 10,000 messages. By contrast, the heatmap for D1 (Figure 5b) evidences that the tree backbone—where LCAs lie—is formed by nodes on the left side of the network, which are also the most congested in a centralized solution (Figure 5a). The two proxies selected by D2 (Figure 5c) are the CNC (0,0) and the node with coordinates (5,6): this confirms our previous observation that D2 both uses different paths than D1 and achieves a better load balancing.

In the second scenario, we consider a network of 100 nodes (hence, 10 producers) and vary the number of consumers such that ≥1 consumers correspond to each producer.

**Figure 6:**
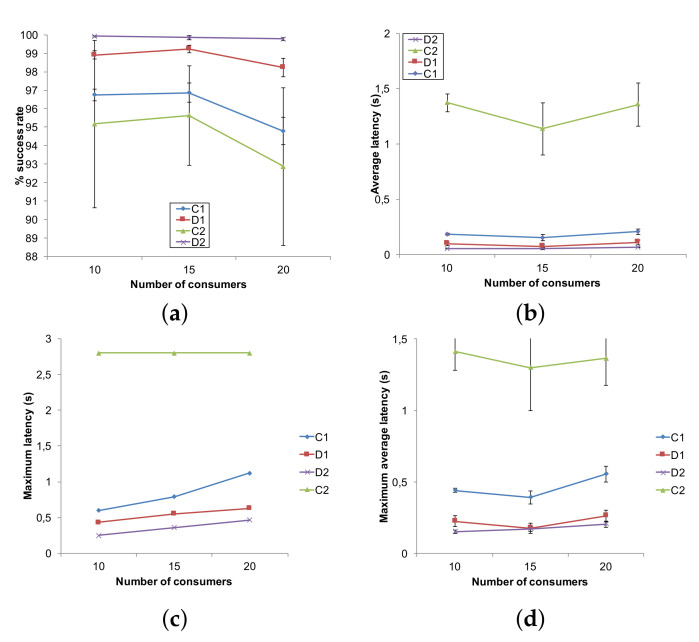
Performance for variable number of consumers. (**a**) Success rate; (**b**) average latency; (**c**) maximum latency; (**d**) maximum average latency.

The results confirm what was observed before: while proxies in D2 allow to maintain a success rate (Figure 6a) very close to 100% (99.785% with 20 consumers), the other solutions—and in particular the centralized ones—suffer as the higher traffic, which produces either collisions (in the case of CSMA/CA), or longer queues and message drop (in the case of TDMA). The average latency, maximum latency and maximum average latency, which are shown in Figure 6b–d respectively, are in accordance to what was observed in the first scenario. This time, the network size is constant while consumers become more dense. This is why the values are almost constant, with a small decline for 15 consumers where possibly some consumer is nearer to the CNC than with 10 consumers. The D2 method succeeds in fulfilling the latency threshold with an average latency of at most 66 ms for the case of 20 consumers. As far as the maximum experimented latency is concerned, in C2 it is constant and equal to 2.8 s, determined by the consumer furthest from the CNC. By contrast, the distributed solutions achieve maximum latency of 0.62 s for D1 and 0.46 s for D2, respectively (both with 20 consumers), showing their adaptability to the positions of both producers and consumers. It is worth noting that D2, while able to guarantee an average latency comparable to the configured threshold, provides no guarantee for the performance observed by the worst consumer; hence, the maximum is largely above the threshold. The maximum latency of 0.46 s is obtained with 20 consumers, while in the first scenario the maximum latency was 0.40 s for a network of 180 nodes.

## 6. The Test Cases

### 6.1. First Test Case

**Figure 7:**
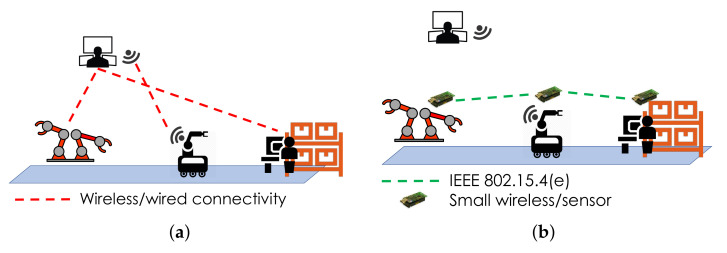
First test case set-up. (**a**) Backbone network operational; (**b**) backbone network not operational.

The set-up of the first test case at the manufacturing experimentation facility is shown in Figure 7a. A mobile robot is responsible for fetching objects to a robotic bi-manipulator. The objects are located at a set of shelves, where a human operator is responsible for manually loading the objects on the mobile robot. Both the robot and the operator are aware of which object is currently needed at the bi-manipulator, as there is a centralized wireless or wired communication infrastructure, coordinated by a CNC, and the data can be sent and received through the communication links. However, due to harsh conditions in several industrial environments, it is not unusual for the main centralized operational network to go offline, for a variety of reasons. When there is a situation like this in the current scenario, the mobile robot and human operator cannot be aware of which object is currently needed at the bi-manipulator, which in turn results in a failure of the production process. In order to address this problem, we suggested the adoption of a distributed data management approach. More specifically, we employed a secondary, lightweight data distribution layer operating using direct communications between the involved nodes, such that data exchange can survive disruptions of the main network infrastructure. We implemented it by using small, low-cost wireless sensor motes. The proof of this concept was showcased by placing three motes in the network as shown in Figure 7b: one on the bi-manipulator, one on the mobile robot and one on the set of shelves. The motes used were IEEE 802.15.4 enabled, a fact that renders them compatible with typical industrial networking protocols, such as IEEE 802.15.4e and WirelessHART. This test case is demonstrating that we can achieve high fault tolerance and reliability with a low-cost approach.

### 6.2. Second Test Case

**Figure 8:**
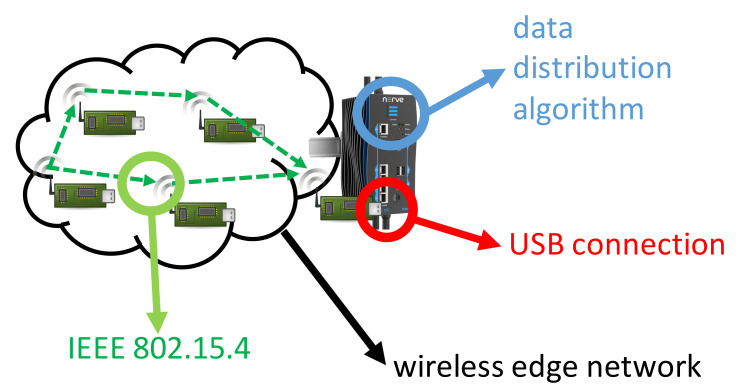
Second test case set-up.

The core concept of the second test case is regarding how we can use smart data distribution at the manufacturing experimentation facility, using an industrial fog node as CNC so as to achieve digital shop-floor reliable data distribution with scalable sensing performance. A fog node is a local server located in the industrial facility premises, which provides localized, cloud-like services to the factory environment. This is therefore an intermediate configuration between the fully centralized one relying on remote cloud services and the fully distributed one experimented in the first use case. With respect to the first use case, this solution will provide a reliable central point for control, at an additional cost in terms of IT infrastructure equipment. Our purpose is not to compare such solutions, but to showcase that smart data distribution schemes can work in a multitude of decentralized configurations. A typical scenario at the manufacturing experimentation facility involves the integrated fog node to control and communicate with various devices of the shop-floor. Reliable data exchanges between the various devices regarding their status (e.g., sensed temperature levels) at any given time should be possible, regardless the operational conditions of the shop-floor. In order to address this, we implemented the integration of distributed data management on a fog node (MFN 100) provided by TTTech. More specifically, we employed the lightweight data distribution layer and implement it by using small, low-cost wireless sensor motes. The proof of this concept was demonstrated by adopting the data management layer introduced in [24] and by placing four motes in the network as shown in Figure 8: two acting as data producers and two acting as data proxies (distributed nodes caching the data) as instructed by the algorithm in [24]. Again, the motes used were IEEE 802.15.4 enabled. The existence of multiple data producers and proxies guarantees that even in the case of failures, the data can still be delivered at the fog node seamlessly. This test case is demonstrating that we can achieve high data redundancy and efficiency with a low-cost approach.

### 6.3. Scalability Performance

**Figure 9:**
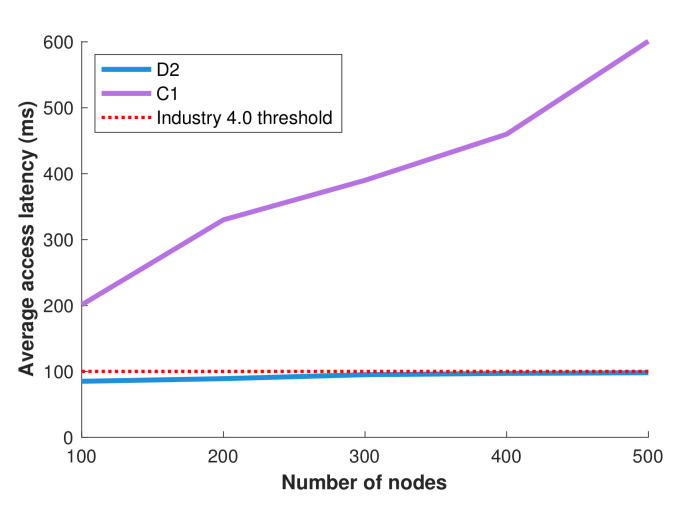
Scalability performance in simulations.

The manufacturing experimentation facility gives us an important ability to test the methods on real conditions and derive useful indications. However, at the same time, it does not allow us to perform larger-scale, or variable experiments, easily and fast. For this reason, and in order to test the scalability of smart data distribution in industrial environments, we developed a simulation model based on the manufacturing experimentation facility layouts. Figure 9 displays the results on the average data access latency for two different alternative approaches, for different numbers of nodes in the network. Specifically, the first approach is the entirely centralized approach C1 which delivers the data to the nodes requesting them through the CNC. The second approach employs our smart distributed data management D2 and uses proxies so as to distributively allocate the data across the network. The red points represent the values for the maximum latency threshold (100 ms) provided by the industrial operator. We can see that the efficient management of proxies provided by the smart data distribution process results in a better performance compared to the entirely centralized alternative. The average latency achieved by smart data distribution respects the typical Industry 4.0 constraints and always remains lower than the data access latency threshold (red line in Figure 9). This simulative result can be explained also intuitively: the dynamic proxy selection process is able to adapt to the underlying latency requirements and arrange the data distribution process in such a way that the corresponding hard constraints are respected.

## 7. Open Practical Problems

The setup and execution of the simulations of Section 5 have brought to light some problems that had not emerged during the design phase, as there was no related material in the literature. Once these problems have been analyzed, possible future developments can be proposed for improvement, to try to solve the problems that arose and be able to collect additional data to evaluate from different network architectural solutions.

During the simulation setup, there emerged a problem linked to the proxy selection algorithm. Specifically, two critical issues emerged, concerning respectively (i) the real-time computation of proxies in method D2, to cope with dynamically joining and leaving consumers; and (ii) a novel channel scheduling policy for TDMA, adequate to deal with the observed queueing saturation and buffer overflow problems. In this section, we discuss both aspects, paving the way to the presentation of the future work introduced in Section 8.

As far as proxy computation is concerned, in the simulation preparation phase, we realized that it has an exponential complexity and for this reason, in the simulations in which the number of consumers was varying, we had to limit the number of consumers to 20, because the estimated time for the calculation of proxies in a network of 100 nodes with 25 consumers was highly impractical: about 12 h. By observing the algorithmic code, we realize that the exponential complexity is caused by the search phase of a new proxy, during which all possible pairs between consumers and proxy candidates are considered. If we indicate as *S* the set of network nodes, $S_{c}$ the set of consumers and *k* the number of proxies that will be found from the algorithm at the end of its execution, the number of combinations that must be calculated is equal to:

$$\sum_{i=1}^{k}\left(|S|-i\right)*{\left(i+1\right)}^{S_{c}}$$

During the simulations, the network architecture that uses the proxy selection algorithm to choose in which nodes to store the data generated by the producers is proved to be the best. The exponential complexity of the algorithm makes it impractical to use in a real context, unless it is a small network. In the type of industrial wireless networks which are composed of hundreds of nodes with poor energy and computational capacity, a constrained node can be unable to perform this algorithm. As a result, the only way to use the distributed network architecture that calculates the proxies with this algorithm is to run the offline algorithm before making the network operational and then insert hardcoded information on the nodes. In doing so, we need to consider that we will have a drop in the performance of the network as soon as one of the proxies goes offline, because none of the consumers associated with this proxy can receive the data to which it is interested in. Furthermore, there would be no node in the network able to re-examine the proxy selection algorithm to calculate a node that takes the place of the fault proxy.

A second problem, which became apparent during the analysis of the results, is the size of the MAC layer queues for the TDMA protocol. As already said for the problem analyzed in the previous paragraph, it is necessary to consider that the network we are working with is a LWPAN. It is therefore unthinkable to have “infinite” queues because the memory of the network nodes is very limited. It is therefore essential to find the right compromise between the size of the queues and the data generation rate to prevent nodes from being in the condition of having to start dropping messages.

## 8. Future Work

The simulations carried out have shown very interesting results. At this point, it would be advisable to carry out simulations with a dynamic network, in which the nodes can move or in which in any case there may be nodes that ask to be added to the network after the initial setup. Measurements similar to those made in simulations should also be made, in a real network, so as to be able to validate the actual precision of the simulator and check if problems arise that cannot exist in the simulator, such as interference caused by the environmental noise of an industrial environment.

Considering the problems related to the complexity of the proxy selection algorithm, it could be interesting to try to optimize the code, given the excellent results found during the simulations. We need to create a solution that is as simple as possible because it will require even lower energy consumption and few computational capabilities. Furthermore, the algorithm should be remodeled to become dynamic; in fact, it was designed to be executed only during the creation of the network, while it would be necessary for the proxies to be recalculated when certain events occur, such as the shutdown of a node or the arrival of a new consumer, so as to continue guaranteeing the initial average latency constraints. We could also think about the development of other algorithms which, instead of being based on the average latency between proxies and consumers, could select the proxies, for example, according to certain energy constraints in order to try to reduce the consumption of the nodes as much as possible.

The results of WirelessHART performance have not shown extreme efficiency due to the problem of devising an efficient channel scheduling policy. However, one of the standards that is being talked about more lately in the industrial wireless field is 6TiSCH, which uses TSCH as a MAC layer protocol [36]. TSCH uses channel slotting as well, but slots may either be used according to a CSMA/CA policy, or be reserved for unicast/broadcast communication from a node to one or all of its neighbours. Furthermore, TSCH includes the concept of “bundle” as a virtual full-duplex channel between a pair of nodes, formed by all the slots reserved for communications between them and supplying them with a certain bandwidth. It might be interesting to implement it in OMNET++ in order to investigate whether the dual policy of channel access might be leveraged to overcome the difficulties encountered with WirelessHART. In fact, based solely on what we experienced with WirelessHART in our simulations, it is a bit difficult to grasp the efficiency of solutions such as 6TiSCH over architectures with CSMA which have been proven efficient in a simulation environment.

Finally, a solution should be found for the problem of the saturation of queues in TDMA: a protocol that, depsite having a non-optimal latency, can guarantee a success rate of 100% would be very useful. To solve this problem, for example, we could revise the node scheduling algorithm which, as it was created, offers a fairly low uplink time but fails to have good results in the downlink, since the superframe must be traversed “reversely”. In fact, WirelessHART requires that the nodes furthest from the gateway be scheduled first: this translates into a superframe with a structure that facilitates the uplink, since there will often be consecutive slots to travel the path toward the gateway, but to go from the gateway to a node, the superframe cannot travel backward and therefore it may take an entire superframe to make a single hop in the downlink. This is one of the reasons why messages pile up in queues. To try to solve the problem, we could try new coloring and scheduling techniques, as shown in [35], or try to implement hybrid channel access techniques, which combine CSMA and TDMA and choose which of the two methods to use depending on the traffic in the network [37].

## 9. Conclusions

In this paper, we propose decentralized data distribution supported by a hierarchical and multi-tier network structure. The proposed solution combines local and decentralized management with centralized decisions to efficiently use the available network resources and meet the requirements of Industry 4.0 applications. Contrary to the recent theoretical and abstract simulative comparisons, we analyze and compare the characteristics of four protocol stacks for data distribution in wireless industrial networks, built mainly from proposed standards, and we evaluate the performance of the four methods via a detailed simulation analysis. The building blocks that we used in order to compose the protocol stacks of the different industrial data distribution methods (both centralized and decentralized) are WirelessHART, RPL and proxy selection. We implemented the methods in OMNeT++ and we evaluated their performance via a detailed simulation analysis. We demonstrated that the careful selection of a limited set of proxies for data caching in the network can lead to increased data delivery success rate and low data access latency. Finally, we demonstrated two test cases in a smart factory context. First, we showed the collaboration between robotic elements and wireless data services. Second, we showed the integration with an industrial fog node which controls the shop-floor devices. We reported selected results in much larger scales, obtained via simulations.

## Figures and Tables

**Table 1:** Comparison of the reported related works.

Paper	Methodological	Simulative	Deployment
Current	✓	✓	✓
[19]	✓	-	-
[20]	✓	-	-
[21]	✓	-	-
[22]	-	✓	-
[23]	-	✓	-
[24]	-	✓	-
[25]	-	-	✓
[26]	-	-	✓
[27]	-	-	✓
[28]	-	-	✓

**Table 2:** Layout of the compared methods.

	C1	D1	D2	C2
**Data cache**	Netw. Contr.	LCAs	Proxies	Netw. Contr.
**APP**	publish–subscribe			
**NET**	RPL non-st.	RPL st.	source routing	
**MAC**	CSMA/CA			WirelessHART
**PHY**	802.15.4 (2.4 GHz)			
